# Quality indicators for the acute and long-term management of anaphylaxis: a systematic review

**DOI:** 10.1186/s13601-017-0151-1

**Published:** 2017-05-24

**Authors:** Sangeeta Dhami, Aadam Sheikh, Antonella Muraro, Graham Roberts, Susanne Halken, Monserat Fernandez Rivas, Margitta Worm, Aziz Sheikh

**Affiliations:** 1 Evidence-Based Health Care Ltd, Edinburgh, UK; 2 UCL, London, UK; 3 Food Allergy Referral Centre Veneto Region, Department of Women and Child Health, Padua General University Hospital, Padua, Italy; 4 The David Hide Asthma and Allergy Research Centre, St Mary’s Hospital, Newport Isle of Wight, NIHR Respiratory Biomedical Research Unit, University Hospital Southampton NHS Foundation Trust, Southampton, UK; 5 Faculty of Medicine, University of Southampton, Southampton, UK; 6 Hans Christian Andersen Children’s Hospital, Odense University Hospital, Odense, Denmark; 7 Hospital Clínico San Carlos - Jefe del Servicio de Alergia, Madrid, Spain; 8 Chartie-Universitatsmedizin, Berlin, Germany; 9 Allergy and Respiratory Research Group, Asthma UK Centre for Applied Research, Usher Institute of Population Health Sciences and Informatics, The University of Edinburgh, Edinburgh, UK

**Keywords:** Allergy, Anaphylaxis, Guidelines, Implementation research, Indicators, Outcomes, Quality of care, Standards

## Abstract

**Background:**

The quality of acute and long-term anaphylaxis management is variable and this contributes to the poor outcomes experienced by many patients. Clinical practice guidelines have the potential to improve outcomes, but implementing guideline recommendations in routine practice is challenging. Quality indicators have the potential to support guideline implementation efforts.

**Objective:**

To identify quality indicators to support the acute and long-term management of anaphylaxis.

**Methods:**

We conducted a systematic review of the literature that involved searching Medline, EMBASE and CINAHL databases for peer-reviewed published literature for the period 1 January 2005–31 December 2015. Additionally we searched Google for grey and unpublished literature. The identified indicators were descriptively summarized against the most recent international anaphylaxis guidelines (i.e. those produced by the European Academy of Allergy and Clinical Immunology) and critically evaluated using the Agency for Healthcare Research and Quality’s criteria for indicator development.

**Results:**

Our searches revealed 830 publications, from which we identified five sources for 54 indicators addressing both acute (n = 27) and long-term (n = 27) management of anaphylaxis. The majority of indicators were developed through expert consensus with relatively few of these having been formally piloted or tested to demonstrate that they could discriminate between variations in practice and/or that they were sensitive to change.

**Conclusions:**

There is a need for a comprehensive set of quality indicators for anaphylaxis management. We have however identified some indicators for the acute and long-term management of anaphylaxis that could with relatively little additional work support efforts to translate guideline recommendations into clinical care.

**Electronic supplementary material:**

The online version of this article (doi:10.1186/s13601-017-0151-1) contains supplementary material, which is available to authorized users.

## Background

Anaphylaxis is a “severe, life-threatening generalized or systemic hypersensitivity reaction” [1, 2] that is responsible for considerable morbidity and, in some cases, mortality. The quality of emergency and ongoing care for patients experiencing and/or with a history of anaphylaxis is variable and this contributes to the poor outcomes (e.g. high risk of recurrent episodes of anaphylaxis) seen [3]. In an attempt to standardize care, and thereby improve outcomes, a number of governments and professional bodies have developed clinical practice guidelines [4–7]. These aim to provide front-line clinicians with simple, concise, evidence-based recommendations for clinical care. Whilst undoubtedly a welcome development, there is a growing body of evidence demonstrating that guidelines often prove challenging to implement in routine clinical care [8]. To support this implementation process, attention is increasingly focusing on the need to develop tools that can help clinicians implement key recommendations and monitor progress with implementation efforts [9].

Quality standards and indicators are potentially important tools designed to help clinicians and healthcare organisations assess the quality of care being provided against agreed evidence-based recommendations [9]. These are now being used across a number of disease and clinical areas, but we are unaware of these currently being routinely used at scale in relation to anaphylaxis.

We are developing evidence-based tools to support translation of key anaphylaxis recommendations into clinical practice and in order to inform this process we undertook a systematic review to identify existing quality indicators for anaphylaxis and identify gaps where there is a need for further development.

## Methods

### Overview of methods, registration and reporting

We conducted a systematic review of the literature that involved searching for published and unpublished literature. It is registered in the PROSPERO database with registration number CRD42016035381. We reported findings using the principles advocated in the PRISMA guidelines [10] (Additional file 1).

### Search strategy

 We developed a highly sensitive search strategy to identify papers on standards and/or quality indicators for anaphylaxis. This involved searching Medline, EMBASE and CINAHL databases for peer-reviewed published literature, and the Google database for searching grey literature published during the period 1 January 2005–31 December 2015. No language restrictions were employed. Our search terms are detailed in the Appendix.

### Inclusion criteria

We were interested in publications reporting on indicators for measuring the quality of acute and long-term care of anaphylaxis in patients of any age. We did not specify any criteria on how these were developed and there was therefore no study filter employed in selecting papers.

### Selection of indicators

Two reviewers independently selected manuscripts against the pre-specified inclusion criteria. Disagreements were resolved through discussion with arbitration by a third reviewer, where necessary.

### Data extraction

Two reviewers independently extracted indicator data onto a customized data extraction sheet. Disagreements were resolved through discussion; a third reviewer arbitrated in instances where agreement could not be reached. Where available, we also extracted data on how these indicators were developed, whether they had been tested and if they had been used in experimental contexts to demonstrate that they could capture improvements in the quality of care.

### Quality assessment of indicators

The quality of these indicators was then assessed against the criteria detailed using the four stage quality indicator process recommended by the Agency for Healthcare Research and Quality (AHRQ), namely:

1. Development: Identifying candidate indicators through a literature review and/or discussion with experts;
2. Implementation: Testing of candidate indicators, introducing them into software etc.;
3. Maintenance: Indicators need to be regularly checked and, if necessary, updated to keep abreast of latest developments; and
4. Retirement processes: Indicators need to be assessed at periodic intervals for relevance and in order to assess if they need to be discontinued [11].

We contacted the authors of these development tools for further clarification, if necessary.

### Data synthesis

We then mapped available indicators against the various recommendations in the most recent international anaphylaxis guidelines, namely those produced by the European Academy of Allergy and Clinical Immunology (EAACI) [12], identifying areas of overlap and gaps, and making an overall assessment of whether any particular indicator was considered appropriate for use in routine clinical practice. Available indicators were traffic-light color coded with green indicating that the indicators were suitable/nearly suitable for routine use as they had undergone the AHRQ process, amber indicating the need for some additional work, and red indicating the need for a substantial amount of additional underpinning work as most of the stages suggested by AHRQ had not been followed.

## Results

### Characteristics of included studies

**Fig. 1:**
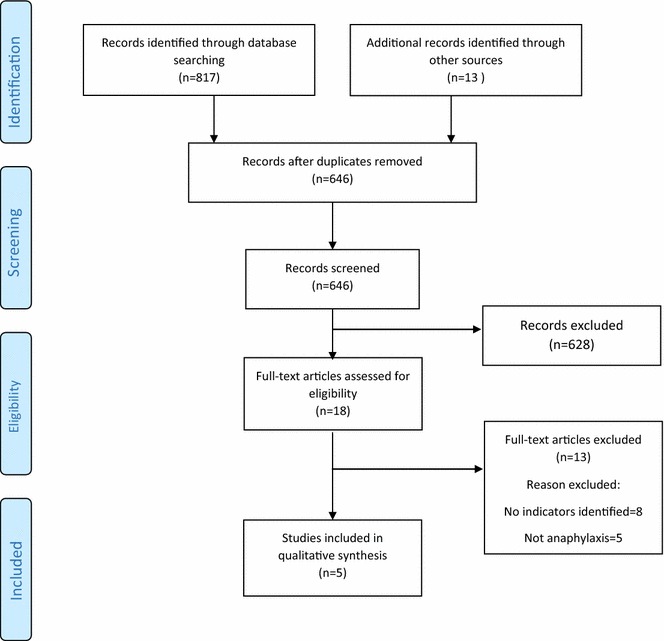
PRISMA flow diagram for anaphylaxis indicators

Our searches identified 830 studies, of which five satisfied our inclusion criteria (see Fig. 1) [12–16]. The five sources of indicators are detailed in Table 1. In total, 54 individual indicators were identified: 27 for the acute management of anaphylaxis and the remaining 27 for longer-term management. Indicators for the acute and longer-term management of anaphylaxis were identified by four of the five sources [12, 14–16]. Two sources of indicators only focused on children and young people [16, 17], and one focused solely on children attending Emergency Departments (ED) for the acute management of anaphylaxis [17].

**Table 1:** Source of indicators for the acute and long-term management of anaphylaxis

Author, year, country	Title	Indicators for the acute management of anaphylaxis	Indicators for the long-term management of anaphylaxis	No of indicators
European Academy of Allergy and Clinical Immunology (EAACI), 2014, Europe	Anaphylaxis: guidelines from the European Academy of Allergy and Clinical Immunology	Yes	Yes	24
Levy M, 2008, UK	Audit of self-administered injectable adrenaline prescription in primary care	Yes	Yes	6
National Institute for Health and Clinical Excellence (NICE), 2011, UK	Anaphylaxis clinical audit tool implementing NICE guidelines	Yes	Yes	8
Royal College of Paediatrics and Child Health (RCPCH), 2011, UK	RCPCH Allergy Care Pathways Project Audit criteria	Yes	Yes	9
Stang AS, et al., 2013, Canada	Quality indicators for high acuity pediatric conditions	Yes	No	7

Geographically, three sets of indicators were developed in the United Kingdom (UK) [14–16], the fourth was developed in Canada [17] and the fifth was pan-European in origin [12].

### Assessment of indicators against AHRQ criteria

Table 2 summarizes our assessment of the quality of the indicators against each of the four criteria stipulated by AHRQ.

**Table 2:** Assessment of indicators against AHRQ criteria

Reference	Identification of candidate indicators			Assessment of candidate indicators					Implementation			Maintenance	Retirement
	Literature review	Conceptual model	Expert engagement	Initial spec	Second literature review	Panel review	Risk adjustment	Empirical analysis	Coding	Testing	User documentation		
EAACI	No	Yes	Yes	No	No	No	No	No	No	No	No	No	No
Levy	No	Yes	Yes	Unclear	No	No	No	No	Yes	Yes	Yes	No	No
NICE	No	Yes	Yes	Yes	No	Unclear	Unclear	No	No	Unclear	Unclear	No	No
RCPCH	No	Yes	Yes	No	No	No	No	Unclear	No	Unclear	Unclear	No	No
Stang	Yes	Yes	Yes	Yes	Unclear	Yes	Yes	Yes	Yes	Yes	Yes	No	No

- 1. Measure development
  The EAACI indicators [12] were derived from clinical guidelines in relation to key recommendations. The Levy indicators [14] were developed through expert consensus. The National Institute of Health and Clinical Excellence (NICE) indicators were derived from relevant guideline recommendations [15]. The Royal College of Paediatrics and Child Health (RCPCH) indicators were derived from a care pathway for children with suspected anaphylaxis [16]. The Stang indicators [17] were the only ones that had been developed through the stages suggested by AHRQ, namely formal processes to identify and assess indicators; furthermore, these were developed using National Quality Framework (NQF) measure evaluation criteria [19].
- 2. Implementation
  The EAACI indicators [12] did not have any formal implementation assessment. The Levy indicators [14] are freely available for use from http://www.guideline-audit.com/adrenaline/audit_specification.php and had been successfully implemented in a number of UK general practices with the opportunity for benchmarking quality of care. NICE [15] had a generic implementation team and created a range of implementation tools, but it was unclear if the ability to implement these indicators in practice had been formally assessed. The RCPCH [16] give no mention of an implementation strategy. The Stang indicators were operationalized and tested in an ED setting [17].
- 3. Maintenance
  None of the indicators had plans for formal maintenance checks.
- 4. Retirement
  There were no plans for retirement of indicators, although EAACI [12], NICE [15] and the RCPCH [16] stated that they had established processes for the periodic review of their clinical guidelines/pathways.

### Mapping of indicators against guideline recommendations

The EAACI Guidelines [12] made 16 recommendations on the acute management of anaphylaxis and indicators were developed by EAACI for all of these recommendations (Table 3). Six of these recommendations also had indicators identified from the other sources.

**Table 3:**
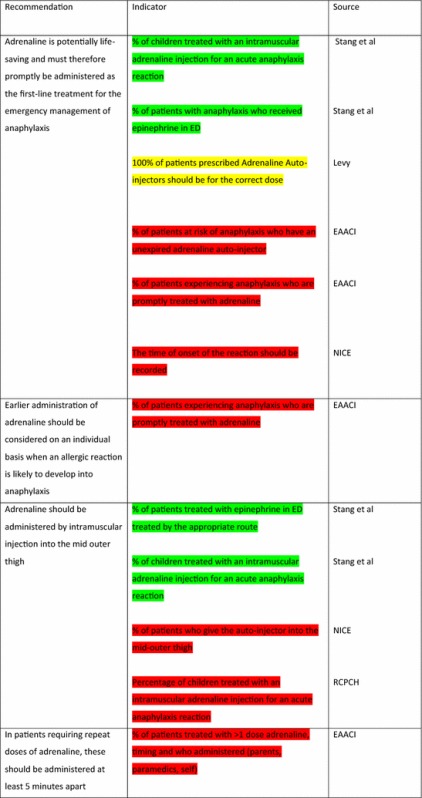

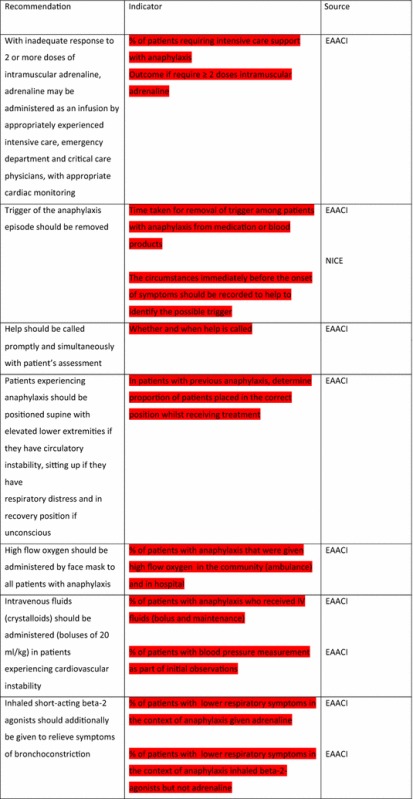

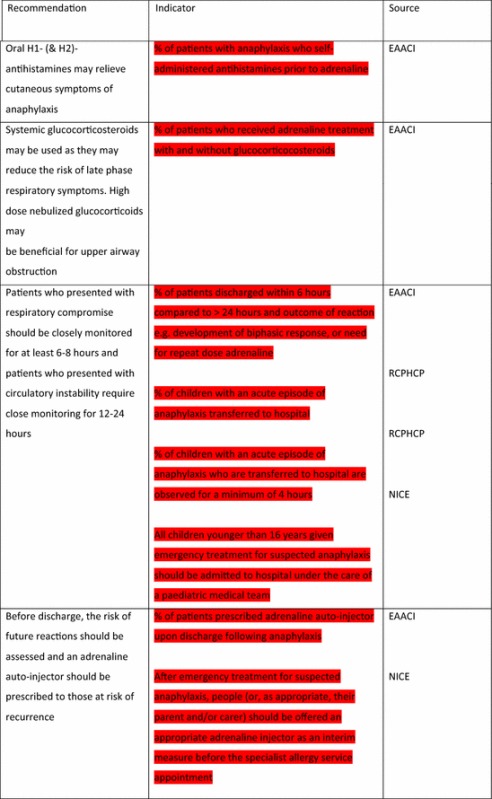

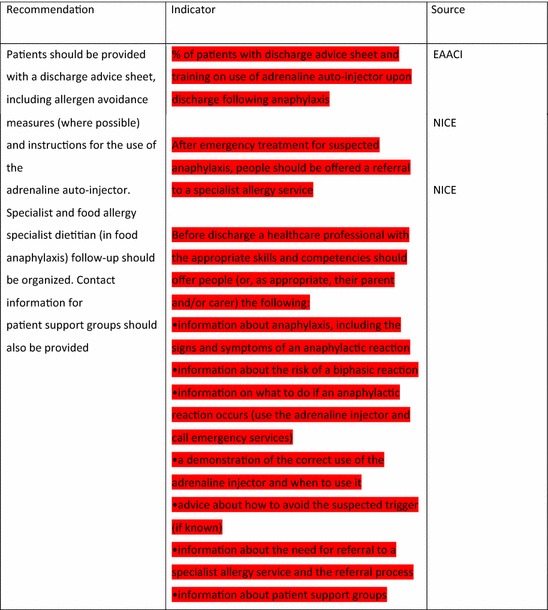
Indicators for the acute management of anaphylaxis mapped to EAACI recommendations with assessment of indicator quality

For the longer-term management of anaphylaxis, EAACI made eight recommendations and indicators were developed by EAACI for all of these (Table 4). Additional indicators from other sources were identified for five of these recommendations.

**Table 4:**
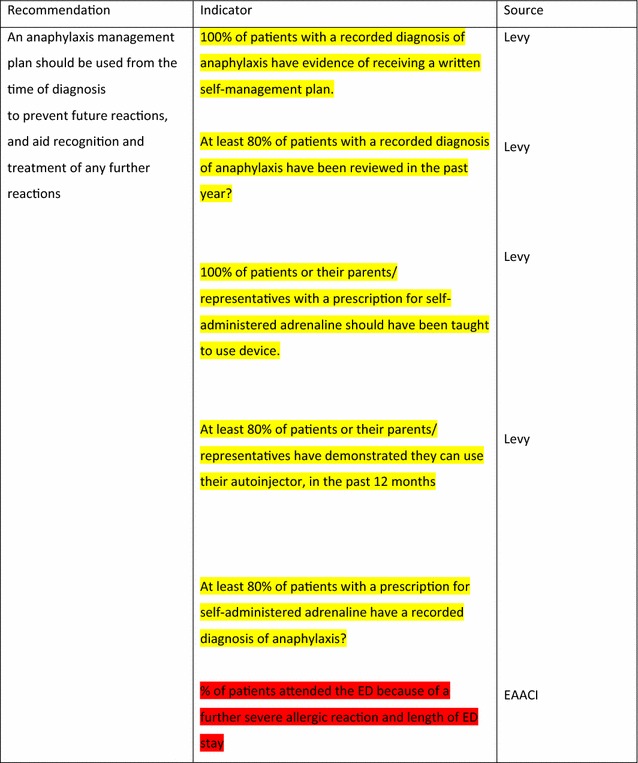

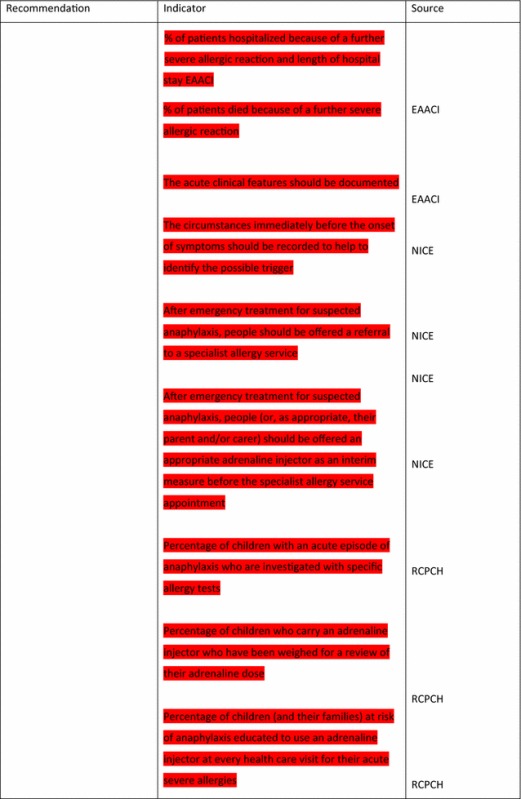

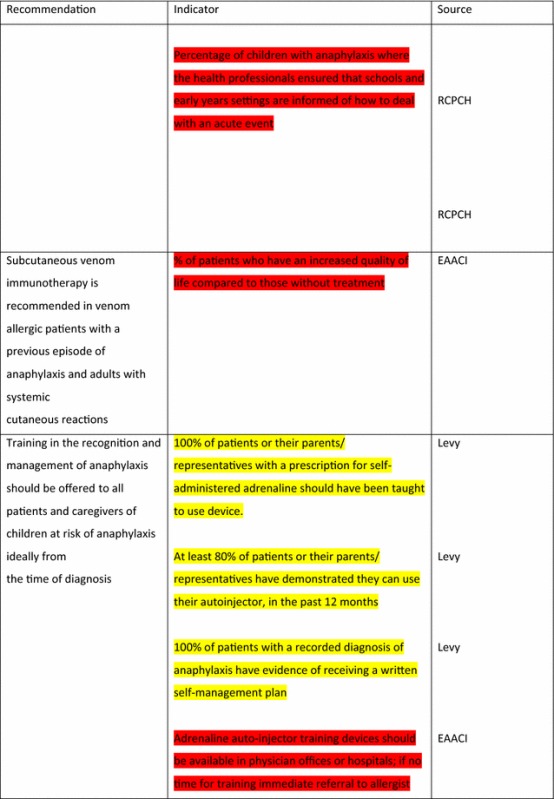

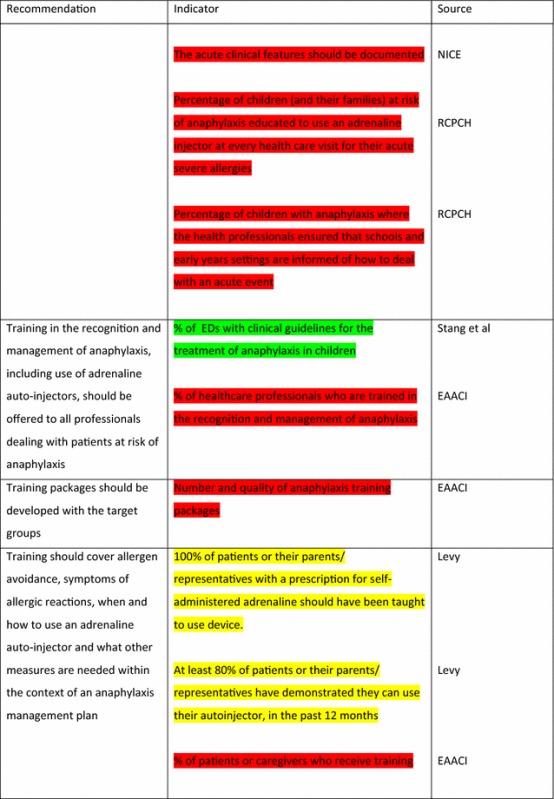

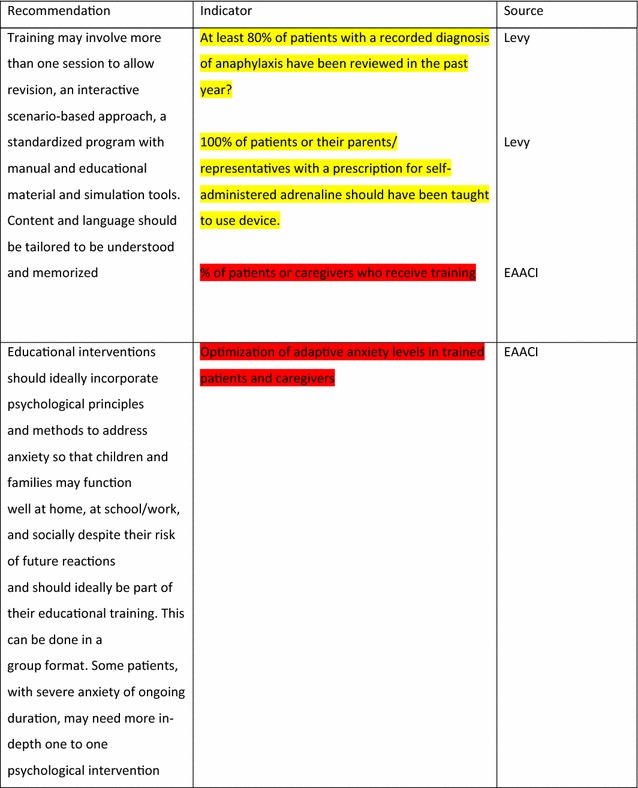
Indicators for the longer-term management of anaphylaxis mapped to EAACI recommendations with assessment of indicator quality

Green, amber and red show which indicators have been developed according to AHRQ criteria, green being the closest and red the furthest

Tables 3 and 4 have been colour coded according to their compliance with the AHRQ criteria to show which indicators are fit-for-purpose. Green identifies indicators that have been developed according to AHRQ principles and are ready to implement, red shows the indicators that need more developmental work before they can be implemented and amber falls between the two. These tables show that the Stang et al. [17] (coded green) and Levy [14] (coded amber) indicators could with relatively modest effort be rendered fit-for-purpose; gaps still however remained in relation to both acute and long-term management (coded red) where considerable development work is still required.

## Discussion

### Statement of principal findings

This study has demonstrated that there are now candidate quality indicators covering many aspects of the acute and long-term management of anaphylaxis. Only a few of these have however undergone the four stages of development recommended by AHRQ, namely implementation and maintenance and none of them have considered decisions on the maintenance or retirement of quality indicators [11]. Further work is therefore needed before any of these can be recommended for routine use in clinical practice [17]. That said, the indicators developed by Stang et al. [16] for acute management of anaphylaxis and those developed by Levy [14] for long-term management could be rendered fit-for-purpose with relatively modest additional effort. EAACI should therefore consider undertaking this work and adopting these indicators. Other areas in relation to both acute and long-term management require much more development work and evaluation.

### Strengths and limitations

The key strengths of this work are that we used systematic review methods to identify relevant literature, formally considered the appropriateness of the methods to develop and deploy these indicators using the four stage process recommended by the AHRQ [11] and then systematically mapped these against the recent EAACI anaphylaxis guidelines [5].

The limitations of this work also need to be considered. This includes the possibility that we failed to identify relevant literature and indicators, although we tried to minimize this risk by not having any restriction of languages on our searches, searching grey literature and by contacting a panel of experts. There may also have been experiences of using these indicators that have not yet found their way into the peer-reviewed or grey literature. This issue could be further investigated through, for example, contacting electronic health record and software vendors to see which if any have been computed and with what results.

### Interpretation in the light of other published literature

Anaphylaxis, in comparison to other disease areas, is relatively undeveloped in terms of quality indicators [18]. For example, NICE has developed indicators for a number of disorders—particularly long-term conditions—that have been used to incentivize improvements in care through the UK Quality and Outcomes Framework (QOF) [19, 20]. Examples of areas in which these have been used include asthma, atrial fibrillation, blood pressure and cancer care [21]. Similarly, in the US indicators are in widespread use in hospital practice focusing, for example, on re-hospitalization of patients within 30 days of discharge, which can be used to penalize hospitals [22, 23]. By imposing financial penalties for those with the highest readmission rates and thus penalizing those with poor levels of care, the hope is to improve the quality of care delivered [24].

### Implications for policy, practice and research

Indicator development, implementation testing, and maintenance and retirement considerations should be seen as integral to the process of producing guidelines as this will maximize the chances of translating guideline recommendations into routine clinical practice and thereby improve outcomes. Quality indicators can improve this translational process through associated financial incentives and penalties as noted above, but they can also be used in more subtle ways through, for example, benchmarking efforts, supporting audit cycles and quality improvement initiatives. These comparative processes, particularly if they involve financial incentives and fines or reputational damage, need to be undertaken with care and with appropriate case mix adjustment, if appropriate [25].

Key next steps are for a multi-stakeholder group to formally consider these existing candidate indicators, chose between existing indicators, propose alternative indicators where considered necessary, develop additional indicators to fill the recommendation gaps, and then undertake formal field work to support implementation efforts. In due course, plans also need to be put into place to consider indicator maintenance and retirement related issues. The AHRQ framework can prove useful to guide this process [11].

## Conclusions

Indicators were identified for all of the recommendations made in the EAACI Anaphylaxis Guidelines, though none of these satisfied all four criteria specified by AHRQ. There are some indicators, particularly in relation to acute management, which would require relatively little effort to render them fit-for-purpose. We also identified some indicators, which may prove suitable in relation to assessing the quality of long-term anaphylaxis care. Other indicators, however, require much more developmental work. To progress this work, stakeholders now need to consider the findings from this review and then undertake additional formative work to ensure that there are a range of suitable indicators that have been both appropriately developed and demonstrated to work in practice to achieve the desired outcome, namely helping to assess the quality of anaphylaxis care delivered to patients.

## Appendix

### Search strategy 1: MEDLINE and EMBASE

1. anaphylaxis/
2. anaphyl*.mp.
3. ((acute or severe or major or serious or life threatening or fatal*) and (allerg* or hypersensiti*)).mp.
4. hypersensitivity immediate/
5. exp food hypersensitivity/
6. respiratory hypersensitivity/
7. exp drug hypersensitivity/
8. ((food or egg? or nut? or peanut? or milk or wheat or drug? or respiratory or asthma* or sting* or venom*) adj3 (allerg* or hypersensiti*)).tw.
9. ((allerg* or hypersensiti*) adj5 reaction*).tw.
10. or/1–9
11. quality indicators.mp. or exp Quality Indicators, Health Care/
12. quality standard.mp.
13. “Process Assessment (Health Care)”/or clinical best practice.mp.
14. clinical audit.mp. or exp Clinical Audit/
15. patient experience.mp.
16. (quality and outcomes framework).mp.
17. or/11–16
18. 10 and 17

### Search strategy 2: CINAHL

(anaphylaxis or anaphylaxis management) AND (quality indicators or quality standard or clinical audit or patient experience).

### Search strategy 3: Google Scholar

Free key word search “anaphylaxis management and quality indicators 2005–2015.

## Additional file

**Additional file 1.** PRISMA checklist.
